# A balancing act: how whole-genome doubling and aneuploidy interact in human cancer

**DOI:** 10.18632/oncotarget.28374

**Published:** 2023-04-26

**Authors:** Kavya Prasad, Uri Ben-David

**Keywords:** cancer, cancer genetics, whole-genome doubling, chromosomal instability

**Figure 1:**
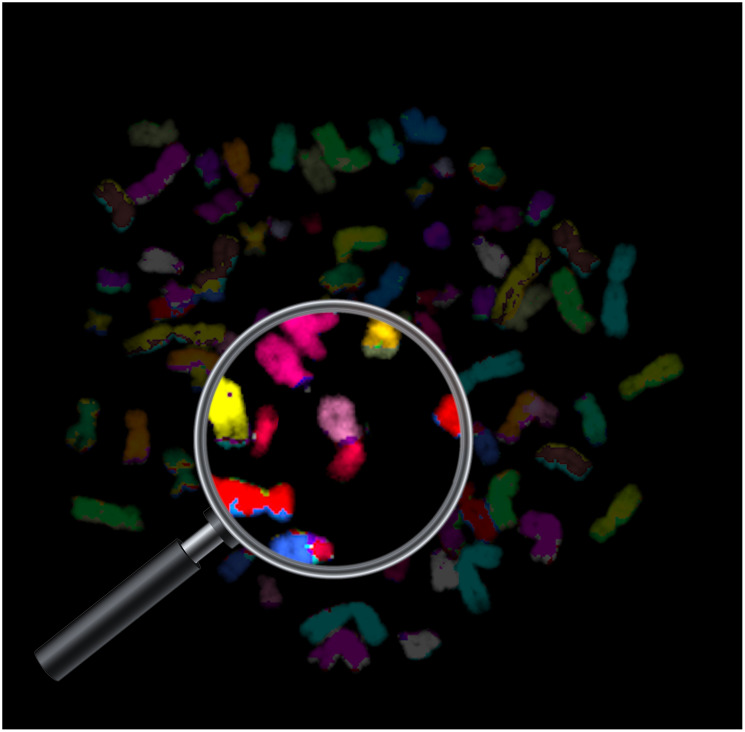
Whole-genome duplication (WGD) occurs in approximately third of human tumors, and is associated with increased chromosomal instability and aneuploidy. Prasad et al. analyzed WGD in human primary tumors and cell lines, demonstrating that WGD increases aneuploidy formation rates and aneuploidy tolerance, and plays an important role in shaping the aneuploidy landscape of human cancers. Shown is a multicolor fluorescence *in situ* hybridization (mFISH) image of a cancer cell that has been through WGD. The magnification focuses on a chromosomal aberration, evident by the existence of multiple colors within the same chromosome. Image created by Sharon Tsach and Uri Ben-David.

Aneuploidy, or an abnormal chromosome copy number, is a characteristic feature of cancer, which plays an important role in cancer initiation and progression [1]. Aneuploidy prevalence patterns are tissue-specific, with different chromosomes gained or lost across cancer types [1–3]. Whole-genome duplication (WGD), also known as whole-genome doubling, occurs in nearly a third of human tumors, usually at the early stages of tumorigenesis [4, 5]. It is known that tumors that have undergone WGD are more permissive to aneuploidy, but whether WGD also affects aneuploidy patterns has remained an open question (Figure 1).

To address this intriguing question, we recently analyzed 5,586 clinical tumor samples that had not undergone WGD (WGD-) and 3,435 tumors that had (WGD+) from The Cancer Genome Atlas (TCGA), across 22 tumor types [6]. WGD- and WGD+ tumors showed distinct aneuploidy patterns; WGD+ tumors were more chromosomally unstable and were more permissive to aneuploidy, presenting not only more aneuploidies in general but also a wider variety of events. Chromosome loss was more common in WGD+ than in WGD- tumors, suggesting that genome doubling might “buffer” the detrimental effect of losing DNA content. In addition, WGD+ tumors had an inclination towards whole-chromosome aneuploidies, as opposed to chromosome-arm changes that were more prevalent in WGD- tumors, indicating distinct dominant mechanisms for aneuploidy formation. Surprisingly, when we examined the interactions between pairs of aneuploidy events, we found that the patterns of co-occurrence and mutual exclusivity were quite different. In rare cases, genetic interactions that were co-occurring in one group became mutually exclusive in the other group within the same tumor type. Similar differences between WGD- and WGD+ tumors were found also when examining the co-occurrence patterns of three, four or five chromosomes at a time. Together, these results suggest that the fitness of specific karyotypes is altered by WGD [6].

We were able to successfully validate several of our findings in human cancer cell lines, as well as in data from patient-derived organoids (PDOs) and xenografts (PDXs), demonstrating the generalizability of our observations and the relevance of common cancer models for the study of WGD [6]. Finally, we quantified aneuploidy and chromosomal instability in isogenic human colon cancer cell lines before and after WGD induction, and were able to experimentally validate the main conclusions from the tumor analysis, supporting a causal role for WGD in shaping the aneuploidy landscapes of human cancer. We note that these experiments were not powered to assess the associations between specific aneuploidies, which remain to be experimentally validated in future studies.

Our study brings up several open questions for future exploration. One important question is whether different methods of tetraploidization might influence aneuploidy landscapes differently. The cell lines used in our study were generated through cytokinesis failure, but other processes, such as cell fusion and endoreduplication, can also cause WGD, and may have distinct impacts on aneuploidy formation and on the fitness value of specific karyotypes. Another related line of research would be to further study the effect of different selection pressures on the evolution of karyotypes. WGD increases the cellular tolerance for a wider spectrum of aneuploidies, but specific karyotypes may prove advantageous under unique conditions and hence get selected for. In other words, the differential impact of WGD on the evolution of specific karyotypes may depend on additional factors beyond the tissue type, such as co-existing mutations, immune surveillance, stage of tumorigenesis and environmental cues. WGD-aware large-scale cancer genomics analyses are required to shed light on these questions.

We also speculate that looking into the patterns of intra-chromosomal arm-level versus whole-chromosomal aneuploidies, may allow us to identify chromosome arms that drive cancer progression. For example, in ovarian cancer, as well as several other tumor types, gain of chromosome 20 is a common aneuploidy. Whereas in WGD+ tumors the entire chromosome is often gained, in WGD- tumors the gain of chromosome arm 20q is sometime associated with a gain and sometimes with a loss of the reciprocal chromosome arm 20p [6]. This may suggest that 20q is the driving force underlying the recurrence of the whole chromosome gain.

In summary, our recent study shows that WGD contributes to aneuploidy formation in human tumors in both qualitative and quantitative ways. Hence, we propose that the WGD status of the tumor should be taken into account when examining the tumorigenic role of individual aneuploidies or aneuploidy patterns. In general, WGD should be considered in the study of aneuploidy landscapes in human cancers.
